# Triterpene Acid (*3*-*O*-*p*-Coumaroyltormentic Acid) Isolated From Aronia Extracts Inhibits Breast Cancer Stem Cell Formation through Downregulation of c-Myc Protein

**DOI:** 10.3390/ijms19092528

**Published:** 2018-08-26

**Authors:** Hack Sun Choi, Su-Lim Kim, Ji-Hyang Kim, Hong-Yuan Deng, Bong-Sik Yun, Dong-Sun Lee

**Affiliations:** 1 Department of Biotechnology, College of Applied Life Science, Jeju National University, Jeju 63243, Korea; choix074@jejunu.ac.kr (H.S.C.); ksl1101@naver.com (S.-L.K.); seogwi12@naver.com (J.-H.K.); hongyuandeng@jejunu.ac.kr (H.-Y.D.); 2 Subtropical/tropical organism gene bank, Jeju National University, Jeju 63243, Korea; 3 Aroma Biotechnology Center, Jeju National University, Jeju 63243, Korea; 4 Division of Biotechnology, College of Environmental and Bioresource Sciences, Chonbuk National University, Gobong-ro 79, Iksan 54596, Korea; bsyun@jbnu.ac.kr

**Keywords:** breast cancer stem cells (CSCs), *3*-*O*-*p*-coumaroyltormentic acid, mammospheres, c-Myc

## Abstract

Cancer stem cells (CSCs) are drug-resistant and radiation-resistant cancer cells that are responsible for tumor progression and maintenance, cancer recurrence, and metastasis. Targeting breast CSCs with phytochemicals is a new paradigm for cancer prevention and treatment. In this study, activity-guided fractionation from mammosphere formation inhibition assays, repeated chromatographic preparations over silica gel, preparatory thin layer chromatography, and HPLC using aronia extracts led to the isolation of one compound. Using ^1^H and ^13^C 2-dimensional nuclear magnetic resonance (NMR) as well as electrospray ionization (ESI) mass spectrometry, the isolated compound was identified as *3*-*O*-*p*-coumaroyltormentic acid. This compound inhibits breast cancer cell proliferation and mammosphere formation in a dose-dependent manner and reduces the CD44^high^/CD24^low^ subpopulation and aldehyde dehydrogenase (ALDH)-expressing cell population as well as the expression of the self-renewal-related genes *CD44*, *SOX2*, and *OCT4.* *3*-*O*-*p*-Coumaroyltormentic acid preferentially reduced the protein levels of c-Myc, which is a CSC survival factor, by inducing c-Myc degradation. These findings indicate the novel utilization of *3*-*O*-*p*-coumaroyltormentic acid for breast cancer therapy via disruption of c-Myc protein, which is a CSC survival factor.

## 1. Introduction

Aronia (*Aronia melanocarpa*) berries, also known as black chokeberries, have recently gained great popularity among consumers primarily owing to their high health benefits [1]. Aronia contains many bioactive compounds, including anthocyanin, carotenoid, fatty acids, flavonoids, phenolic compounds, and vitamins [2,3]. Specifically, the anthocyanin content in aronia berries is much higher than that in other plants and is known for its antioxidant, anti-inflammatory, and antiaging effects [4]. The health-promoting potential of aronia is widely recognized, and the results of preclinical and clinical studies support the health benefits of aronia [5,6]. Recently, aronia has received increasing attention due to its antioxidant, anti-inflammatory, and anticancer activity [1,7].

Among women, breast cancer is a common cancer and a major cause of cancer-related death [8]. Breast cancer is still a highly dangerous disease because of cancer recurrence and metastasis. Cancer stem cells (CSCs) were first identified in myeloid leukemia [9], and later were reported as a subpopulation of various solid tumors, including breast, brain, colon, ovarian, pancreatic, and prostate cancer [10]. CSCs are also known as tumor-initiating cells and cancer stem-like cells. In addition, much evidence has shown that many types of cancer (including breast cancer) originate from CSCs, which compose a small population within the tumor [11]. This subpopulation produces the tumor bulk through self-renewal and differentiation [12]. CSCs have multidrug-resistant and radioresistant properties against chemotherapy and radiotherapy, respectively, to eradicate the bulk tumor, which thus results in cancer recurrence and metastasis [13]. Therefore, therapies targeting CSCs are essential in cancer treatment. Recently, the antioxidant activities of blackberry were enhanced by fermentation via lactic acid bacteria [14]. The chokeberry extract may exhibit its anticancer potential by inducing apoptosis and inhibiting invasion via reduced MMP gene expression in U373 cells [15]. *Aronia melanocarpa* juice (AMJ) exerts its chemotherapeutic properties against acute lymphoblastic leukemia by selectively targeting lymphoblast-derived tumor cells [16].

In this study, we chose aronia to target breast CSCs. The anti-CSC activity of aronia extracts against breast cancer was tested, and we isolated the active component that possesses anti-CSC activity in breast cancer by activity-guided fractionation. The purified, active compound, *3*-*O*-*p*-coumaroyltormentic acid, inhibited CSC formation. For the first time, we demonstrate that *3*-*O*-*p*-coumaroyltormentic acid exerts anti-CSC activity against breast cancer through the deregulation of c-Myc expression, which is a CSC survival factor.

## 2. Results

### 2.1. Isolation of the CSC Inhibitor From Aronia

**Figure 1:**
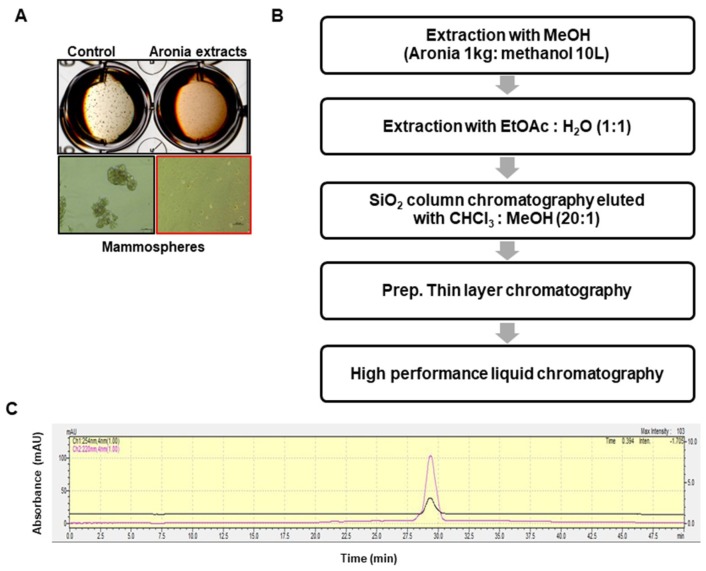
Isolation of the cancer stem cell (CSC) inhibitor from aronia and assessment of the aronia extracts via mammosphere formation assay. (**A**) Assay of mammosphere formation inhibition using aronia extracts. The mammospheres were incubated with aronia extracts or DMSO. MDA-MB-231 cells were treated with aronia extracts or DMSO in CSC culture media for seven days. Images were obtained by microscopy at 10× magnification and were representative mammospheres (scale bar = 100 µm). (**B**) Isolation procedure of the mammosphere-forming inhibitor. (**C**) HPLC chromatogram of the inhibitor isolated from the aronia extracts.

To screen for breast CSC inhibitors from aronia extracts, we performed the mammosphere formation assay using methanol extracts of aronia and observed inhibition of mammosphere formation. We concluded that the methanol extracts contained the CSC inhibitor (Figure 1A). The bioassay-guided isolation procedure is summarized in Figure 1B. The methanol extracts were isolated using ethyl acetate extract, silica gel, preparatory thin layer chromatography (TLC), and HPLC. The purified sample was confirmed using HPLC (Figure 1C).

### 2.2. Structure Determination of the Isolated CSC Inhibitor

**Figure 2:**
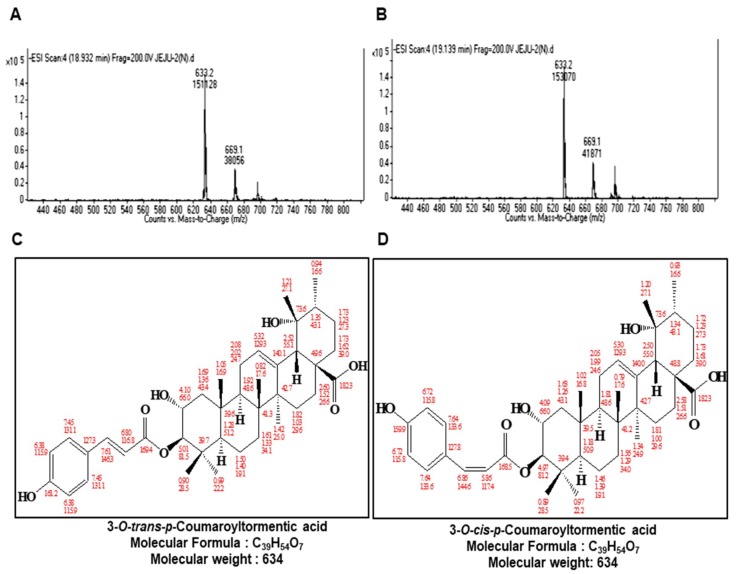
Molecular structure of the CSC inhibitor isolated from aronia. (**A**,**B**) Molecular mass of the CSC inhibitor derived from aronia based on electrospray ionization (ESI) mass spectrometry. (**C**,**D**) Molecular structure of *3*-*O*-*trans*-*p*-coumaroyltormentic acid and *3*-*O*-*cis*-*p*-coumaroyltormentic acid.

The chemical structures of the compounds isolated were determined by mass spectrometry and NMR measurements. The molecular weight of 3-*O*-*trans*-*p*-coumaroyltormentic acid was established as 634 by ESI mass spectrometry, which showed quasimolecular ion peaks at *m*/*z* 635.2 [M + H]^+^ in positive mode and at *m*/*z* 633.2 [M − H]^−^ in negative mode (Figure 2A,B). The ^1^H NMR spectrum of 3-*O*-*trans*-*p*-coumaroyltormentic acid in CD_3_OD exhibited signals due to aromatic methine protons at δ 7.45 and 6.38, which are attributable to a 1,4-disubstituted benzene, and two *trans*-conjugated olefinic protons at δ 7.61 and 6.80 that were identical to signals for *trans*-*p*-coumaric acid. Additional signals in the ^1^H NMR spectrum were typical for a triterpenoid. The 39 carbons in the ^13^C NMR spectrum were evident, and the chemical shifts of the signals due to the triterpene portion were in agreement with those of tormentic acid, suggesting that the structure of 3-*O*-*trans*-*p*-coumaroyltormentic acid is tormentic acid esterified with coumaric acid. All of the proton-bearing carbons were assigned by the HMQC spectrum, and the ^1^H-^1^H COSY spectrum revealed eight partial structures (see Supplementary Materials). Further structural confirmation was performed by assessment of the HMBC spectrum, which showed a long-range correlation from the methine proton at δ 5.01 to the carbonyl carbon of coumaric acid at δ 169.4. Other HMBC correlations were well-matched to coumaroyltormentic acid (Figures S4–S13). Therefore, the structure of the compound was identified as 3-*O*-*trans*-*p*-coumaroyltormentic acid (Figure 2C).

The compound 3-*O*-*cis*-*p*-coumaroyltormentic acid is closely related to 3-*O*-*trans*-*p*-coumaroyltormentic acid in its mass and NMR spectra, suggesting that it is also a triterpene coumaric acid ester. The molecular weight was the same as that of *O*-*trans*-*p*-coumaroyltormentic acid. The ^1^H and ^13^C chemical shifts of this triterpene portion were in agreement with those of *O*-*trans*-*p*-coumaroyltormentic acid. However, 3-*O*-*cis*-*p*-coumaroyltormentic acid differed from *O*-*trans*-*p*-coumaroyltormentic acid in the ^1^H coupling constant and the ^13^C chemical shifts of the coumaroyl moiety. The ^1^H coupling constant of two olefinic protons at δ 6.86 and 5.86 revealed the presence of *cis*-*p*-coumaric acid. Therefore, the structure of this second compound was identified as 3-*O*-*cis*-*p*-coumaroyltormentic acid (Figure 2D).

### 2.3. 3-O-p-Coumaroyltormentic Acid Inhibits Breast Cancer Cell Proliferation and Mammosphere Formation

**Figure 3:**
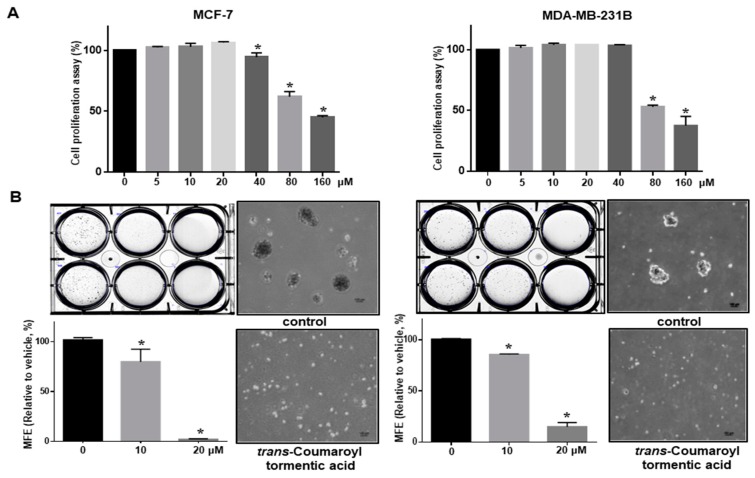

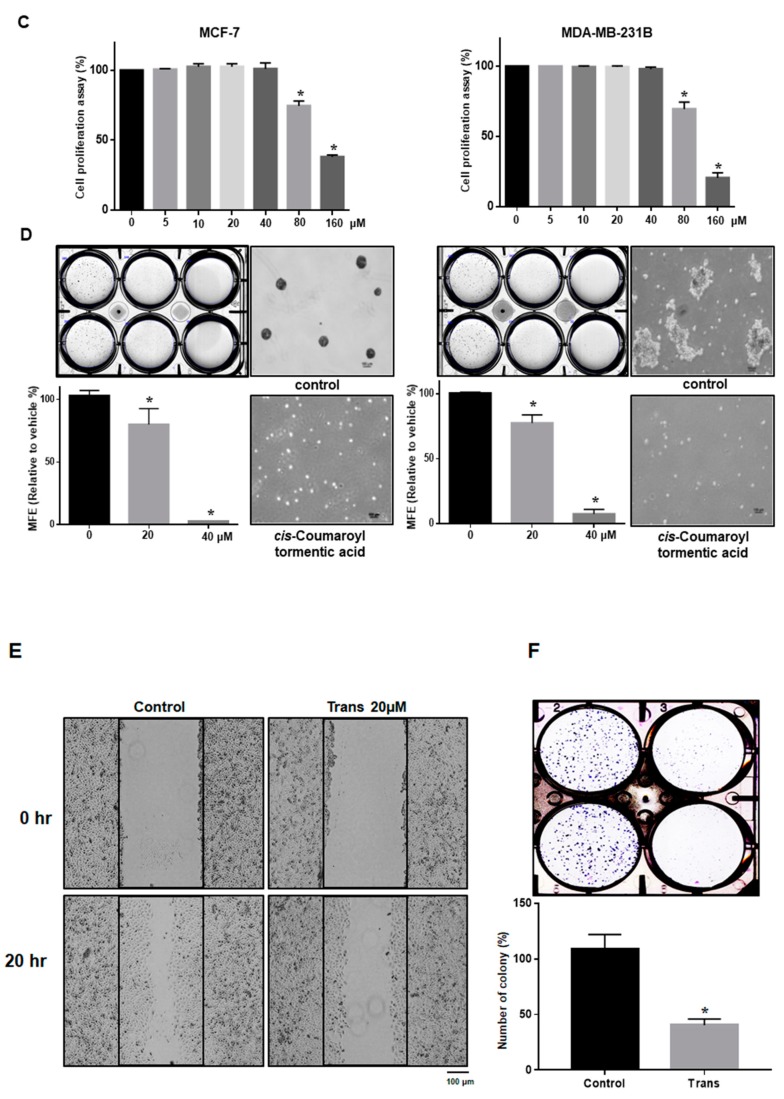
The effect of *3*-*O*-*p*-coumaroyltormentic acid on the viability and mammosphere-forming ability of MCF-7 and MDA-MB-231 cells. (**A**) MCF-7 and MDA-MB-231 cells were treated with increasing concentrations of *3*-*O*-*trans*-*p*-coumaroyltormentic acid for 48 h. The antiproliferative effect of *3*-*O*-*trans*-*p*-coumaroyltormentic acid was measured by the MTS (3-(4,5-dimethylthiazol-2-yl)-5-(3-carboxymethoxyphenyl)-2-(4-sulfophenyl)-2*H*-tetrazolium) assay. (**B**) Effect of *3*-*O*-*trans*-*p*-coumaroyltormentic acid on the formation of mammospheres derived from MCF-7 and MDA-MB-231 cells. The mammospheres were incubated with either *3*-*O*-*trans*-*p*-coumaroyltormentic acid (10 and 20 μM) or DMSO for seven days (scale bar = 100 μm). (**C**) MCF-7 and MDA-MB-231 cells were treated with increasing concentrations of *3*-*O*-*cis*-*p*-coumaroyltormentic acid for 48 h. The antiproliferative effect of *3*-*O*-*cis*-*p*-coumaroyltormentic acid was measured by the MTS assay. (**D**) Effect of *3*-*O*-*cis*-*p*-coumaroyltormentic acid on the formation of mammospheres derived from MCF-7 and MDA-MB-231 cells. The mammospheres were incubated with either *3*-*O*-*cis*-*p*-coumaroyltormentic acid (20 and 40 μM) or DMSO. MCF-7 and MDA-MB-231 cells were treated with *3*-*O*-*cis*-*p*-coumaroyltormentic acid or DMSO in CSC culture media for seven days. Images were obtained by microscopy at 10× magnification and were representative mammospheres (scale bar = 100 μm). (**E**) Effect of *3*-*O*-*trans*-*p*-coumaroyltormentic acid on migratory potential of human breast cancer cells. The wound healing of MDA-MB-231 cells with or without *3*-*O*-*trans*-*p*-coumaroyltormentic acid photographed at 0 and 18 h (scale bar = 100 μm). (**F**) Effect of *3*-*O*-*trans*-*p*-coumaroyltormentic acid on colony formation on human breast cancer cells. The dissociated 1000 MDA-231-MB cells were seeded in six-well plates and treated with an indicated concentration of *3*-*O*-*trans*-*p*-coumaroyltormentic acid and DMSO for seven days. Representative images of colonies were recorded. The data shown represent the mean ± SD of three independent experiments. * *p* < 0.05 vs. DMSO-treated control.

We first examined the antiproliferative effect of *3*-*O*-*trans*-*p*-coumaroyltormentic acid in the human breast cancer cell lines MCF-7 and MDA-MB-231 by MTS (3-(4,5-dimethylthiazol-2-yl)-5-(3-carboxymethoxyphenyl)-2-(4-sulfophenyl)-2*H*-tetrazolium) assay. There was a concentration-dependent inhibition of cell proliferation at ≥40 μM (MCF-7) and ≥80 μM (MDA-MB-231) *3*-*O*-*trans*-*p*-coumaroyltormentic acid after 48 h of stimulation (Figure 3A). These results showed that *3*-*O*-*trans*-*p*-coumaroyltormentic acid effectively inhibits the proliferation of these breast cancer cell lines. To evaluate whether this compound can suppress the formation of tumorspheres or mammospheres, we treated *3*-*O*-*trans*-*p*-coumaroyltormentic acid to primary mammospheres derived from MCF-7 and MDA-MB-231 cells. As shown in Figure 3B, *3*-*O*-*trans*-*p*-coumaroyltormentic acid effectively inhibited the formation of primary mammospheres derived from breast cancer cell lines. Not only did the numbers of mammospheres decline by 90%, the size of mammospheres was also reduced (Figure 3B). In addition, *3*-*O*-*cis*-*p*-coumaroyltormentic acid inhibits cell proliferation at concentrations ≥80 μM (MCF-7 and MDA-MB-231 cells) after 48 h of stimulation (Figure 3C) and the formation of primary mammospheres derived from breast cancer cell lines at 40 μM (Figure 3D). The treatment with *3*-*O*-*trans*-*p*-coumaroyltormentic acid inhibited the migration and colony formation of MDA-MB-231 cells (Figure 3E,F). These results showed that *3*-*O*-*trans*-*p*-coumaroyltormentic acid effectively inhibits mammosphere formation and the various cancer hallmarks (proliferation, migration, and colony formation).

### 2.4. Treatment with 3-O-trans-p-Coumaroyltormentic Acid Decreased the CD44^high^/CD24^low^-Expressing Subpopulation and the Proportion of ALDH-Positive Breast Cancer Cells

**Figure 4:**
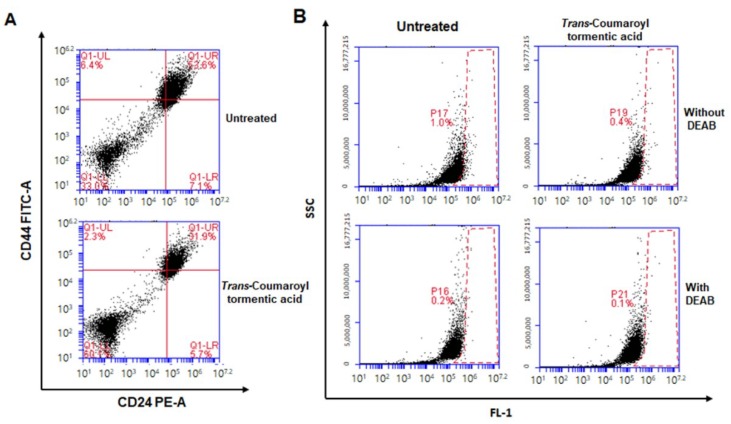
Effect of *3*-*O*-*trans*-*p*-coumaroyltormentic acid on the expression of CSC markers in breast cancer cell line. (**A**) The CD44^high^/CD24^low^ cell population was analyzed by flow cytometry in MDA-MB-231 cells treated with either *3*-*O*-*trans*-*p*-coumaroyltormentic acid (20 μM) or DMSO for two days. For FACS (Flow Cytometer) analysis, 50,000 cells were acquired. Gating was based on binding of the control antibody (red cross). (**B**) The effect of *3*-*O*-*trans*-*p*-coumaroyltormentic acid on the ALDH-positive cell population. MDA-MB-231 cells were treated with either *3*-*O*-*trans*-*p*-coumaroyltormentic acid (20 μM) or DMSO for two days and subjected to the ALDEFLUOR assay and FACS analysis. A set of representative flow cytometer dot plots is shown. The lower panel shows ALDH-positive cells in the presence of the ALDH inhibitor diethylaminobenzaldehyde (DEAB) as a negative control, and the upper panel represents ALDH-positive cells in the absence of DEAB. The ALDH-positive population was gated in the box (red dot line box).

The CD44^high^/CD24^low^ expression pattern as well as aldehyde dehydrogenase (ALDH) expression is a breast CSC marker. We checked the effect of *3*-*O*-*trans*-*p*-coumaroyltormentic acid on the CD44^high^/CD24^low^-expressing and ALDH-positive subpopulations. MDA-MB-231 cells were treated with *3*-*O*-*trans*-*p*-coumaroyltormentic acid for 24 h, and the CD44^high^/CD24^low^-expressing subpopulation in breast cancer cells was measured to investigate the effect of the inhibitor. Treatment decreased the CD44^high^/CD24^low^-expressing subpopulation among breast cancer cells (Figure 4A). MDA-MB-231 cells were treated with this inhibitor for 24 h and subjected to the ALDEFLUOR assay to investigate the effect of *3*-*O*-*trans*-*p*-coumaroyltormentic acid on the proportion of ALDH-positive breast cancer cells; the results indicated that this compound decreased the proportion of ALDH-positive breast cancer cells from 1.0 to 0.4% (Figure 4B).

### 2.5. Effect of 3-O-Trans-p-Coumaroyltormentic Acid on the Protein Expression Levels of c-Myc in Breast CSCs

**Figure 5:**
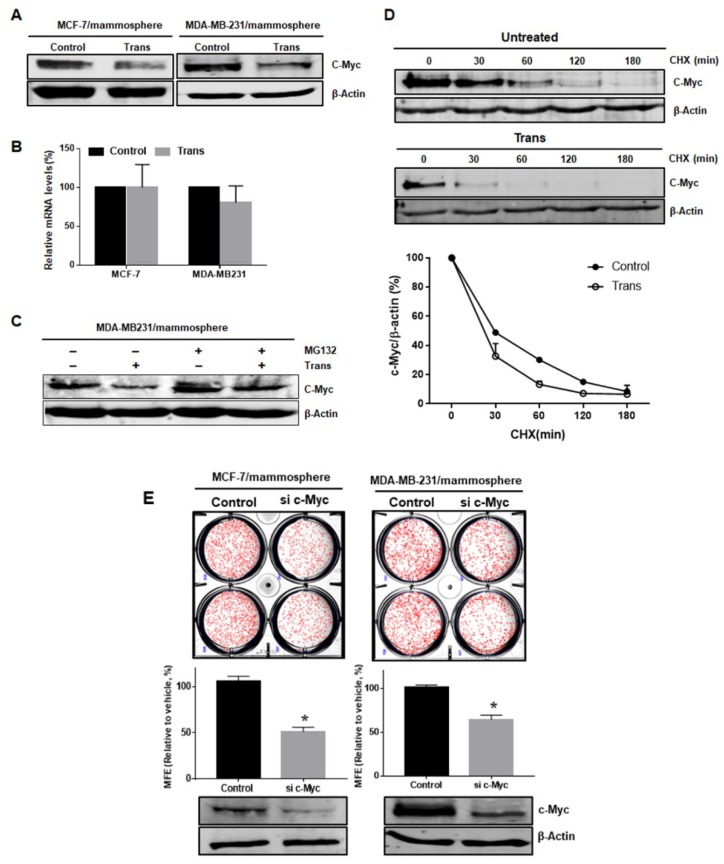
The *trans*-coumaroyltormentic acid (Trans) promotes the proteasome-mediated degradation of c-Myc in breast CSCs. (**A**) Trans reduced the protein level of c-Myc. Cells were treated with Trans (20 μM) for 24 h. Total cell lysates were subjected to Western blot analysis with specific antibodies. β-Actin was used as an internal control. (**B**) Transcriptional expression of c-Myc genes of CSCs was determined in Trans- and DMSO-treated mammospheres using c-Myc-specific primers and real-time PCR. β-Actin served as an internal control. (**C**) Mammospheres were incubated with MG-132 and Trans (20 μM) for 24 h and lysed for Western blot analysis. (**D**) The cycloheximide (CHX) chase assays showing the half-life of c-Myc protein. The mammospheres were treated with CHX at 100 µg/mL for the indicated times. The half-life of endogenous c-Myc protein was measured by Western blot and analyzed. Each point represents the mean ± SD of triplicate experiments. (**E**) Effect of c-Myc protein on mammosphere formation using siRNA knockdown of c-Myc. The mammospheres derived from siRNA-treated cells were cultured for seven days. Images were obtained by microscopy at 10× magnification and are representative mammospheres (scale bar = 100 μm). The data shown represent the mean ± SD of three independent experiments. * *p* < 0.05 vs. DMSO-treated control. All of the immunoblots are representative of more than three independent experiments.

c-Myc is an important oncogene that is required for the maintenance of glioma CSCs [17]. Given the importance of c-Myc in CSC survival, we examined the effects of *3*-*O*-*trans*-*p*-coumaroyltormentic acid on c-Myc expression in breast CSCs. *3*-*O*-*trans*-*p*-coumaroyltormentic acid treatment significantly downregulated the protein expression of c-Myc, but did not reduce the c-Myc transcript levels in breast CSCs (Figure 5A,B). Treatment of cells with the proteasome inhibitor MG132 protected c-Myc from *3*-*O*-*trans*-*p*-coumaroyltormentic acid-induced c-Myc degradation, suggesting that *3*-*O*-*trans*-*p*-coumaroyltormentic acid enhances the proteasomal degradation of c-Myc (Figure 5C). The results of the cycloheximide experiment suggested that *3*-*O*-*trans*-*p*-coumaroyltormentic acid increased the degradation rate of c-Myc protein through an ubiquitin-independent pathway (Figure 5D and Figuge S14).

### 2.6. c-Myc is a Survival Factor for Breast CSC Formation

To confirm the function of c-Myc as a CSC survival factor, we assessed the activity of cells with siRNA-mediated silencing of c-Myc. MDA-MB-231 cells transfected with c-Myc-specific siRNA were shown to have reduced mammosphere formation (Figure 5E). In conclusion, we found that c-Myc is important factor in CSC formation (Figure 5).

### 2.7. 3-O-trans-p-Coumaroyltormentic Acid Inhibits the Expression of Self-Renewal Genes in CSCs and the Proliferation of Mammospheres

**Figure 6:**
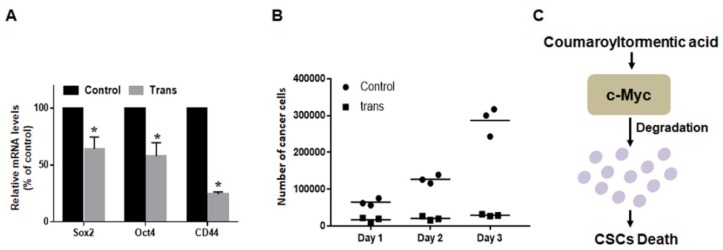
Effect of *3*-*O*-*trans*-*p*-coumaroyltormentic acid on the gene expression of CSC markers and mammosphere growth. (**A**) Transcriptional expression of the CSC markers Sox2, CD44, and Oct4 genes was determined in *3*-*O*-*trans*-*p*-coumaroyltormentic acid-treated and DMSO-treated mammospheres using specific primers for these genes and real-time RT-PCR. β-Actin served as an internal control. (**B**) Effect of *3*-*O*-*trans*-*p*-coumaroyltormentic acid on mammosphere growth. *3*-*O*-*trans*-*p*-coumaroyltormentic acid prevents mammosphere growth. After *3*-*O*-*trans*-*p*-coumaroyltormentic acid and DMSO treatment for two days, the mammospheres were dissociated into a single-cell suspension and plated in a six-cm dish with an equal number of cells. At 24 h after plating, the cells were counted. At two days and three days after replating, cells were counted in triplicate and plotted as the mean value. The data shown represent the mean ± SD of three independent experiments. * *p* < 0.05 vs. DMSO-treated control. (**C**) The proposed model for CSCs’ death through c-Myc degradation (purple dots) by *3*-*O*-*trans*-*p*-coumaroyltormentic acid.

To determine whether *3*-*O*-*trans*-*p*-coumaroyltormentic acid inhibits the expression of self-renewal genes, we examined the expression levels of some self-renewal genes by real-time RT-PCR. Treatment with *3*-*O*-*trans*-*p*-coumaroyltormentic acid decreased the expression of Sox2, CD44, and Oct4 in breast CSCs (Figure 6A). To confirm that *3*-*O*-*trans*-*p*-coumaroyltormentic acid inhibits mammosphere proliferation, mammospheres were treated with *3*-*O*-*trans*-*p*-coumaroyltormentic acid, and cell numbers within the mammospheres were counted. *3*-*O*-*trans*-*p*-Coumaroyltormentic acid induced the cell death of mammospheres, and a lower cell number was observed in *3*-*O*-*trans*-*p*-coumaroyltormentic acid-treated mammospheres. This result indicated that *3*-*O*-*trans*-*p*-coumaroyltormentic acid led to a dramatic decrease in mammosphere proliferation (Figure 6B). Taken together, these results suggest that *3*-*O*-*trans*-*p*-coumaroyltormentic acid inhibits the self-renewal and proliferation of CSCs.

## 3. Discussion

Aronia (*Aronia melanocarpa*), also known as black chokeberries, contain many bioactive compounds, including polyphenols, vitamin C, anthocyanins, phenolic acids, flavanols, and tannins, all of which are natural antioxidants [18]. The antioxidant activities of natural compounds can be increased by fermentation using fungi, yeast, or lactic acid bacteria [19,20]. For the first time, we isolated a breast CSC inhibitor from aronia powder. Activity-guided fractionation and repeated chromatographic separation resulted in the separation of one compound from aronia powder. This is the first report describing how *3*-*O*-*trans*-*p*-coumaroyltormentic acid inhibits breast CSCs.

*3*-*O*-*p*-Coumaroyltormentic acid, triterpene acid, induces apoptotic cell death in the human leukemia HL60 cell line via mainly the mitochondrial pathway by, at least in part, the inhibition of topoisomerase I. Furthermore, it may be a promising lead compound for developing an effective drug for treating leukemia [21]. This compound, a constituent from gold-red apple, has α-glucosidase inhibitory activities [22], and α-glucosidases have served as a useful platform in the discovery and development of drugs targeting obesity and type II diabetes [23].

Breast cancer is a major cause of morbidity and mortality in women. The small population of CSCs in breast cancer tumors are involved in self-renewal and contribute to aggressiveness, drug resistance, and relapse [24]. Many CSCs were isolated from primary patient samples and several cancer cell lines. As there is a limited supply of CSCs derived from primary tissue samples, CSCs from cancer cell lines were selected and studied for CSC research [25].

Our results provide several lines of evidence for *3*-*O*-*p*-coumaroyltormentic acid as an anti-CSC agent for breast cancer therapy. (1) *3*-*O*-*p*-Coumaroyltormentic acid inhibited the proliferation of breast cancer cells. The mammosphere formation of MCF-7 and MDA-MB- 231 cells was inhibited in the presence of *3*-*O*-*p*-coumaroyltormentic acid (Figure 3). Additionally, *3*-*O*-*p*-coumaroyltormentic acid reduced the size and formation of mammospheres derived from primary breast cancer. (2) *3*-*O*-*p*-Coumaroyltormentic acid reduced CD44^high^/CD24^low^ and ALDH-positive subpopulations in MDA-MB-231 cells (Figure 4). (3) *3*-*O*-*p*-Coumaroyltormentic acid reduced the protein level of c-Myc. c-Myc is an essential factor for CSC formation, and *3*-*O*-*p*-coumaroyltormentic acid inhibits the formation of mammospheres by blocking the c-Myc pathway (Figure 5). (4) *3*-*O*-*p*-Coumaroyltormentic acid dramatically decreases mammosphere proliferation. These new findings showed that *3*-*O*-*p*-coumaroyltormentic acid could be a novel anticancer agent for breast cancer therapy by targeting CSCs.

The proto-oncogene c-Myc is a transcription factor that regulates a variety of target genes related to cell growth, differentiation, and apoptosis [26]. c-Myc is a short-lived protein that undergoes ubiquitin-dependent [27] and ubiquitin-independent degradation [28]. MYC inhibition depletes cancer stem-like cells in triple-negative breast cancer, and the central role of c-Myc is the regulation of proliferation and survival of glioma cancer stem cells [17,29]. Our data showed that *3*-*O*-*trans*-*p*-coumaroyltormentic acid induced c-Myc protein degradation through the ubiquitin-independent pathway (Figure 5D and Figure S14), and suggests that the proliferation, growth, and survival of breast cancer stem cells are critically dependent on c-Myc expression and that targeting c-Myc pathways may significantly improve breast tumor therapy.

More recently, a number of studies have found that several triterpenoids can inhibit cancer stem cells. For example, ginsenoside Rg3, a triterpene saponin, inhibits CSCs of colorectal cancer [30] and gedunin, a natural tetranortriterpenoid, inhibits teratocarcinomal (NTERA-2) cancer stem-like cells [31]. The cucurbitacin E as a tetracyclic triterpene, betulonic acid, a pentacyclic lupane-type triterpenoid, and plant-derived triterpenoid tingenin B inhibit cervical cancer cell lines, leukemia stem cells, and breast CSCs, respectively [32,33,34]. Also, triterpene glycosides may be used as adjuvants for the rational design of vaccines against cancer [35]. The phenethyl isothiocyanate, a naturally occurring isothiocyanate, and 8-bromo-7-methoxychrysin, a synthetic derivative of chrysin that is a naturally widely distributed flavonoid, has been shown to possess inhibitory activity against colon CSCs and liver CSCs [36,37].

The pharmacokinetics and pharmacodynamics (PK/PD) modeling approach of a triterpene acid (*3*-*O*-*p*-coumaroyltormentic acid) may provide a conceptual framework for its future clinical evaluation as a dietary chemopreventive agent in humans. Similar phytochemicals of triterpenoid ursolic acid (UA) has been proposed as a potential cancer chemopreventive agent in preclinical and clinical studies. The plasma concentration of UA was determined using a liquid chromatography-mass spectrometry (LC-mass). A biexponential decline in the UA plasma concentration was observed after UA injection. The pharmacodynamics (PD) markers, phase II DM/anti-oxidant genes, increased and peaked approximately 3 h after 20 mg/kg UA treatment phase. UA is effective at inducing various phase II DM/antioxidant genes and inhibiting pro-inflammatory genes in vivo [38]. If severe combined immunodeficient (SCID) mice bearing human breast cancer xenograft was treated with *3*-*O*-*p*-coumaroyltormentic acid, we could measure the concentration in plasma and tissues using LC mass, tumor size, and c-Myc stability. Triterpenoids are ubiquitous in the plant kingdom. Recent evidence supports the beneficial effects of naturally occurring triterpenoids against several types of human cancers. The anticancer potential of triterpenoids and their anti-inflammatory and antiproliferative effects have been discussed both in vitro and vivo models [39]. Both naturally occurred and synthetic derivatives showed chemopreventive and therapeutic effects by using tumor xenograft models. Also, triterpenoids showed chemopreventive and anticancer potentials on various preclinical animal models of colon, breast, prostate, and melanoma cancers [39].

In summary, we report that *3*-*O*-*p*-coumaroyltormentic acid can target breast CSCs as determined by the mammosphere formation, ALDEFLUOR, and mammosphere proliferation assays. We also revealed that *3*-*O*-*p*-coumaroyltormentic acid targets breast CSCs through c-Myc degradation. Our data suggest the possible usage of *3*-*O*-*p*-coumaroyltormentic acid as a new breast cancer chemotherapy.

## 4. Materials and Methods

### 4.1. Reagents 

Silica gel 60A (35–75 microns) (ANALTECH, Newark, DE, USA) was used for column chromatography. Thin layer chromatography (TLC) was performed on a Kieselgel 60 F_254_ plate (MERK, Darmstadt, Germany). The TLC plates were developed with a chloroform–methanol solution (20:1) and visualized using a Spectroline UV lamp (Westbury, NY, USA). Tissue culture plates, including six-well and 24-well ultralow attachment cluster plates, were obtained from Corning. Cell viability was measured using a CellTiter 96^®^ Aqueous One Solution cell proliferation assay kit (Promega, WI, USA). An ALDEFLUOR™ Kit was purchased from STEMCELL Technologies Inc (Vancouver, BC, Canada). *3*-*O*-*trans*-*p*-Coumaroyltormentic acid and *3*-*O*-*cis*-*p*-coumaroyltormentic acid were obtained from ChemFaces (Wuhan, Hubei, China). All other chemicals were obtained from Sigma-Aldrich Co (St. Louis, MO, USA).

### 4.2. Plant Material

Aronia cultivated on Jeju Island, South Korea was purchased from urban farmers (Seogwipo, Jeju, Korea). The black berries were washed with tap water, dried, and ground with a grinder (Hanil, Seoul, Korea). A voucher specimen (No. 2016_010) has been deposited in the Subtropical/Tropical Organism Gene Bank of Jeju National University (Jeju, Korea).

### 4.3. Extraction and Isolation

Dried powder of aronia (1 kg) was extracted with 10 L of methanol, after which the methanol extracts were dried, and the sample was solubilized with 500 mL of methanol. Next, 500 mL of distilled water was added to the mixture, which was sequentially partitioned with equal volumes of ethyl acetate. The ethyl acetate fraction was concentrated and solubilized with 100% methanol. The bioassay-guided isolation procedure is summarized in Figure 1B. The concentrated methanol fraction was added to a silica gel column (25 × 350 mm) and eluted with a chloroform–methanol solution (20:1) (Figure S1). Six fractions were obtained and assessed using the mammosphere formation assay. The #6 fraction showed the most potential to inhibit mammosphere formation and thus was subjected to preparatory TLC (glass plate; 20 × 20 cm) before development in the chloroform–methanol solvent (20:1) using a TLC glass chamber. After development, the plates were removed and dried, and the fluorescence was visualized under UV radiation (UV_254nm_ and UV_365nm_). Individual bands were isolated by scraping off the target area of the silica gel from the glass plate using a scalpel and collecting the gel into a 15-mL conical tube. Methanol (5 mL) was added to isolated gel, and the mixture was centrifuged and dried under vacuum. Each band was tested using the mammosphere formation assay (Figure S2). The active band was subjected to preparatory HPLC analysis, which was performed using a Shimadzu HPLC 20A (Shimadzu, Tokyo, Japan). Chromatographic separation was conducted using a Shim-pack GIS-PREP-ODS 10 × 250 mm C18 column. The sample was prepared and filtered through a 0.2-μm syringe filter for HPLC analysis. The injection volume for HPLC isolation was 500 μL, the flow was 3 mL/min, the column was at room temperature, and the detection was performed at 220 and 254 nm. The mobile phase was composed of water (solvent A) and methanol (solvent B). For gradient elution, solvent B was initially set at 20%, increased 60% at 20 min, and increased to 100% at 40 min. The purified sample was detected at a retention time of 45 min (Figure S3).

### 4.4. Structure Analysis of Purified Sample

Electrospray ionization (ESI) mass spectrometry was conducted on a QTRAP-3200 mass spectrometer (Applied Biosystems, Foster City, CA, USA). Nuclear magnetic resonance (NMR) spectra were obtained on a JEOL JNM-ECA 600 FT-NMR spectrometer at 600 MHz for ^1^H NMR and at 150 MHz for ^13^C NMR in CD_3_OD. Chemical shifts are presented as ppm (δ), with tetramethylsilane as the internal standard. For NMR spectra, two-dimensional NMR such as ^1^H-^1^H COSY, HMQC and HMBC as well as one-dimensional NMR such as ^1^H NMR and ^13^C NMR were employed (Figures S4–S13).

### 4.5. Culture of Human Breast Cancer Cells and the Formation of Mammospheres

The human breast cancer cell lines MCF-7 and MDA-MB-231 were obtained from the Korea Cell Line Bank (KCLB, Seoul, Korea). Both cell lines were grown in Dulbecco’s Modified Essential Medium (DMEM; HyClone, Logan, UT, USA) supplemented with 10% fetal bovine serum (FBS; HyClone), 100 U/mL of penicillin, and 100 μg/mL of streptomycin (HyClone), and were maintained at 37 °C in a humidified incubator containing 5% CO_2_. We used breast cancer cell lines up to 15 passages after purchasing cell lines from KCLB and the cell lines were authenticated by the KCLB and showed >90% similarity in a short tandem repeats (STR) DNA fingerprint profile when compared with the American Type Culture Collection (ATCC) and Korean Cell Line Bank (KCLB) databases. Cells were plated at a density of 1 × 10^6^ cells in 10-cm culture dishes. To establish primary mammospheres, single-cell suspensions of MCF-7 and MDA-MB-231 cells were seeded at a density of (3.5∼4) × 10^4^ and (0.5∼1) × 10^4^ cells/well, respectively, in ultralow attachment six-well plates containing 2 mL of complete MammoCult^TM^ medium (StemCell Technologies; Vancouver, BC, Canada), which was supplemented with 4 μg/mL of heparin, 0.48 μg/mL of hydrocortisone, 100 U/mL of penicillin, and 100 μg/mL of streptomycin. The cells were incubated for seven days in a 5% CO_2_ incubator at 37 °C.

### 4.6. Automated Counting of Mammospheres

After seven days of culture, 8-bit grayscale images of the mammospheres were acquired by placing the cell culture plate on a scanner (Epson Perfection V700 PHOTO, Epson, Tokyo, Japan). Low-resolution images (300 dpi) were loaded using the software program NICE (ftp://ftp.nist.gov/pub/physics/mlclarke/NICE) [40]. For counting, regions of interest (ROIs) were created by choosing the desired number of rows and columns (e.g., 2 × 3 for a six-well plate), and individual ROIs were defined by moving and resizing the provided ROI shapes after selecting the elliptical setting in the NICE program. The background signal of the images was negated using thresholding algorithms, and the selected images were automatically counted. The results of the mammosphere formation assay are reported as the mammosphere formation efficiency (MFE, % of control), which corresponds to the number of mammospheres per well/the number of total cells plated per well ×100, as previously described [40].

### 4.7. Cell Proliferation Assay

A CellTiter 96^®^ Aqueous One solution cell proliferation assay kit was used to measure the proliferation rate of MCF-7 and MDA-MB-231 cells following the manufacturer’s protocol. MCF-7 and MDA-MB-231 cells were grown in a 96-well plate in the presence of *3*-*O*-*p*-coumaroyltormentic acid (0, 5, 10, 20, 40, 80 and 160 μM) for 48 h, and the optical density at 490 nm was determined using a 96-well plate reader (VersaMax ELISA Microplate; Molecular Device, San Jose, CA, USA). Each condition was measured in triplicate.

### 4.8. Flow Cytometric Analysis of CD44 and CD24 Expression

Expression of CD44 and CD24 in MDA-MB-231 cells was determined by FACS analysis. After the harvesting and dissociation of cells using 1× Trypsin/EDTA, one million cells were suspended, labeled with fluorescein isothiocyanate (FITC)-conjugated anti-human CD44 and PE-conjugated anti-human CD24 antibodies (BD) and incubated at 4 °C for 30 min. Then, the cells were washed three times with 1× PBS and analyzed on a flow cytometer (Accuri C6, BD, San Jose, CA, USA).

### 4.9. Clonogenic Assay

MDA-MB-231 cells were seeded at low density in a six-well plate and treated with *3*-*O*-*p*-coumaroyltormentic acid in DMEM media. After 24 h, the media was replaced with fresh media and cultured to grow for seven days. The grown colonies were counted.

### 4.10. Scratch Migration Assay

MDA-MB-231 cells were seeded in a six-well plate and grown at 90% confluency. The scratch was made through the cell layer using a sterile white micropipette tip. After washing with DMEM media, breast cancer was treated with *3*-*O*-*p*-coumaroyltormentic acid or DMSO. Wounded areas were photographed with a light microscope at 10× after 16 h.

### 4.11. Real-Time RT-PCR

The levels of transcripts were determined with a One Step SYBR PrimeScript RT-PCR kit using SYBR Green as a double-stranded DNA specific dye according to the manufacturer’s instructions (Takara, Tokyo, Japan). One-step RT-PCR was carried out with 1 μg of total RNA, 10 μL of 2× One Step SYBR RT-PCR Buffer IV, 1 μL of PrimeScript 1 step Enzyme Mix II, 10 μM of forward primer, and 10 μM of reverse primer for a final volume of 20 μL per reaction. The following primer pairs were used: OCT4, forward primer: AGCAAAACCCGGAGGAGT, reverse primer: CCACATCGGCCTGTGTATATC; SOX2, forward primer: TTGCTGCCTCTTTAAGACTAGGA, reverse primer: CTGGGGCTCAAACTTCTCTC; CD44, forward primer: AGAAGGTGTGGGCAGAAGAA, reverse primer: AAATGCACCATTTCCTGAGA; and β-actin, forward primer: TGTTACCAACTGGGACGACA, reverse primer: GGGGTGTTGAAGGTCTCAAA. The relative mRNA expression levels of the target genes were calculated using the comparative CT method. At least three independent PCR experiments were performed to allow statistical analysis. The PCR expression levels were normalized to those of β-actin, which served as an internal control.

### 4.12. ALDEFLUOR Assay

The ALDEFLUOR assay system offers a novel approach to the identification, evaluation, and isolation of CSCs based on the aldehyde dehydrogenase (ALDH) activity. The active reagent BODIPY (boron-dipyrromethene)–aminoacetaldehyde was added to breast cancer cells, which converted the reagent to fluorescent BODIPY–aminoacetate via ALDH. Cells administered the ALDH inhibitor diethylaminobenzaldehyde (DEAB) were used as a negative control. MCF-7 cells were treated with 20 μM of 3-*O*-*trans*-*p*-coumaroyltormentic acid for 24 h, and the proportion of ALDH-positive cells was analyzed with the ALDEFLUOR assay. ALDH-positive and ALDH-negative cells were sorted using a flow cytometer (Accuri C6).

### 4.13. Western Blotting

Proteins isolated from MCF-7 and MDA-MB-231 mammospheres treated with 3-*O*-*trans*-*p*-coumaroyltormentic acid were separated on a 10% gel with SDS-PAGE and transferred to a polyvinylidene difluoride membrane (Millipore, Billerica, MA, USA). Membranes were blocked in 5% nonfat dry milk in PBS-Tween 20 (0.1%, *v*/*v*) at room temperature for 30 min. The blots were incubated at 4 °C overnight in a blocking solution containing the following primary antibodies: c-Myc (Cell Signaling Technology, Denver, CO, USA) and β-actin (Santa Cruz Biotechnology, Dallas, TX, USA), the latter of which was used as a loading control. After the membranes were washed with PBS-Tween 20 (0.1%, *v*/*v*), they were incubated with IRDye 800CW and IRDye 680RD-conjugated secondary antibodies, and band signals were detected using an ODYSSEY CLx (LI-COR, Lincoln, NB, USA).

### 4.14. Small Interfering RNA (siRNA)

To determine the effect of c-Myc on mammosphere formation, we treated MDA-MB-231-derived mammospheres with human c-Myc siRNA (NM_002467.3; Bioneer, Daejeon, Korea) or scrambled siRNA control (Bioneer). To transfect the siRNA, MDA-MB-231 cells were seeded into six-well plates for 24 h, and transfection was performed using Lipofectamine 3000 (Thermo Scientific, Waltham, MA, USA) according to the manufacturer’s protocol. The level of c-Myc protein was determined by Western blot using anti-c-Myc antibodies and the conditions described above.

### 4.15. Statistical Analysis

All of the data are presented as the mean ± standard deviation (SD). Data were analyzed using Student’s *I*-test. A *p*-value less than 0.05 was considered statistically significant (GraphPad Prism 5 Software).

## 5. Conclusions

Triterpene acid (*3*-*O*-*p*-coumaroyltormentic acid) inhibits mammosphere formation and reduces the CD44^high^/CD24^low^ subpopulation, ALDH-expressing cell population, and the expression of the self-renewal-related genes. The compound reduced the protein levels of c-Myc and indicate the novel utilization for breast cancer therapy via disruption of c-Myc protein, which is a CSC survival factor.

## Supplementary Materials

Supplementary materials can be found at http://www.mdpi.com/1422-0067/19/9/2528/s1.

## Abbreviations

CSCs	Cancer stem cells
AMJ	*Aronia* melanocarpa juice
NICE	NIST’s integrated colony enumerator
ESI	Electrospray ionization
NMR	Nuclear magnetic resonance
COSY	Correlation spectroscopy
HMQC	Heteronuclear multiple-quantum correlation spectroscopy
HMBC	Heteronuclear multiple-bond correlation spectroscopy
ROI	Regions of interest
MFE	Mammosphere formation efficiency
Trans	*trans*-Coumaroyltormentic acid
