# Supplementary material for: Triterpene Acid (3-O-p-Coumaroyltormentic Acid) Isolated From Aronia Extracts Inhibits Breast Cancer Stem Cell Formation through Downregulation of c-Myc Protein
Source: Int J Mol Sci. 2018 Aug 26;19(9):2528. doi: 10.3390/ijms19092528 (PMC6164992; doi:10.3390/ijms19092528)
Supplement: Supplementary file 1 [file ijms-19-02528-s001.pdf]

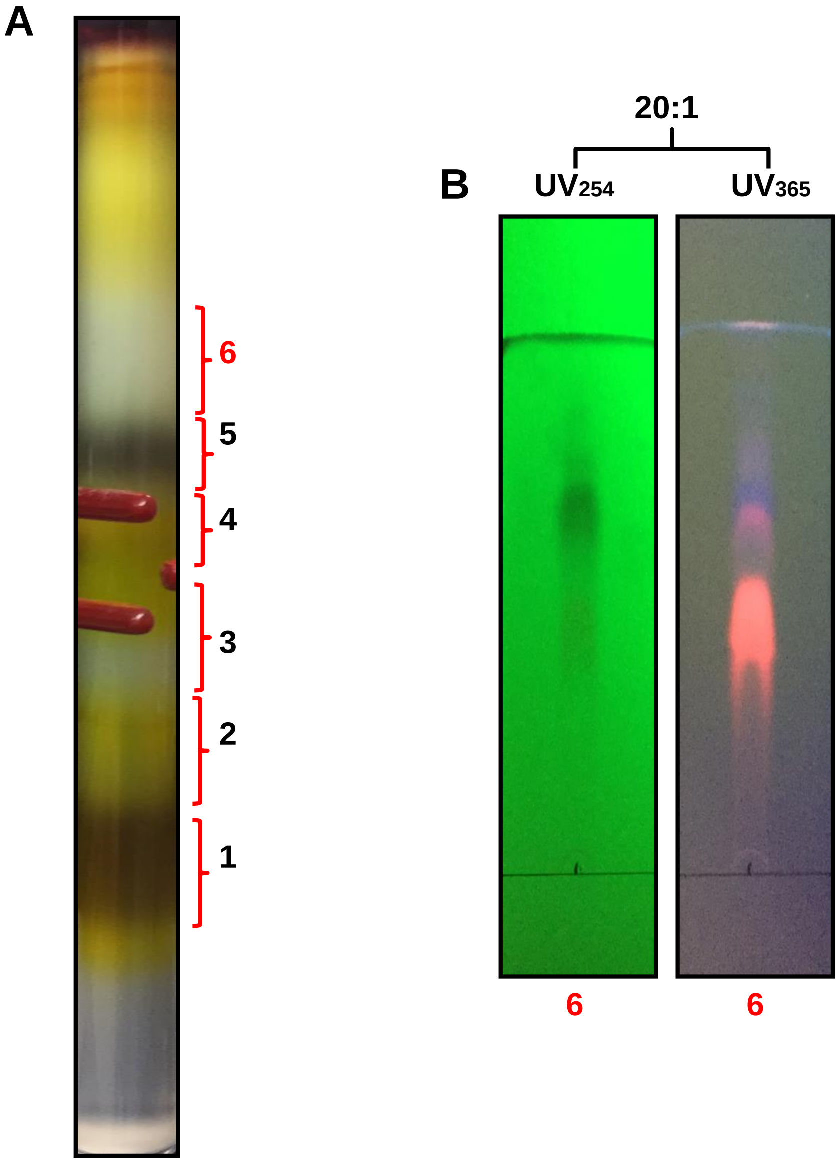

Supplementary Figure S1. Purification procedure of the inhibitor of mammosphere formation derived from aronia extracts using SiO_2 gel chromatography. (A) The sample was isolated using SiO_2 gel chromatography with a solvent mixture [$CHCl_{3:}$ MeOH (20:1)]. (B) TLC plate analysis of a partially purified sample. Active fraction; #6.

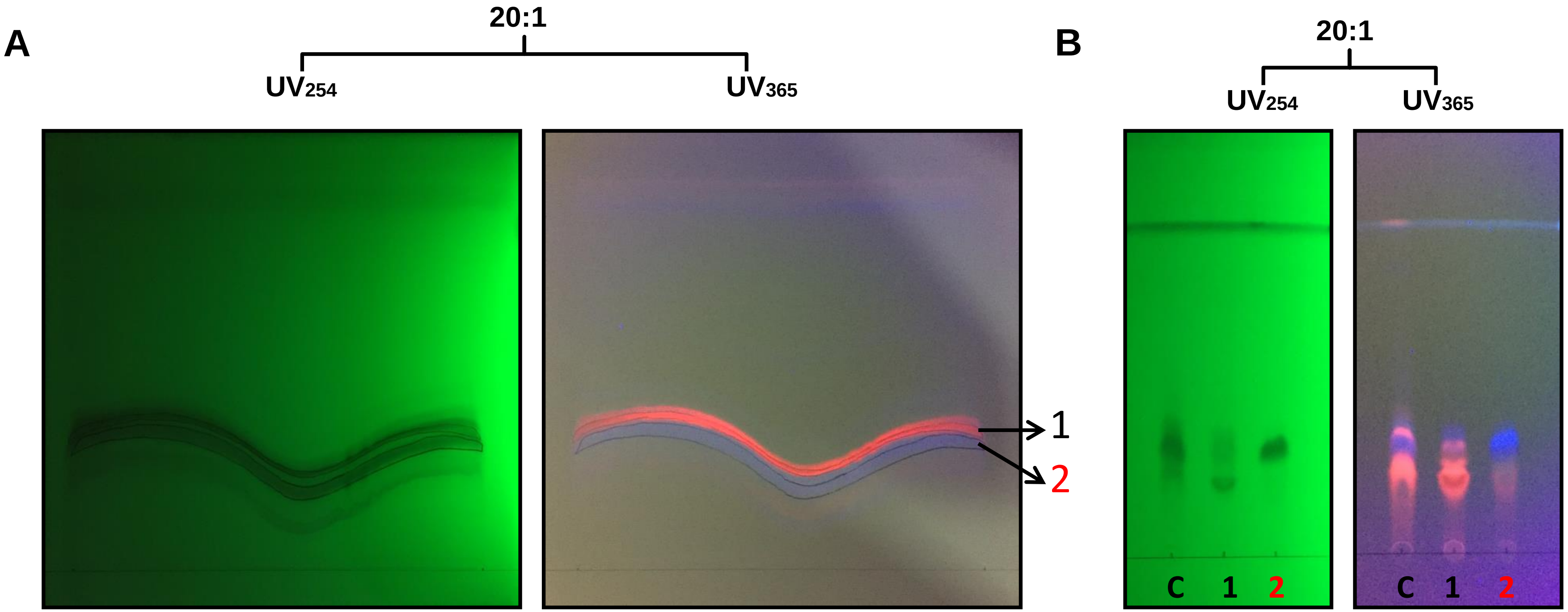

Supplementary Figure S2. Purification procedure of the inhibitor of mammosphere formation from aronia extracts using preparative thin layer chromatography with $CHCl_3$:MeOH (20:1). (A) Preparatory TLC chromatography containing fractions 1 and 2. (B) TLC analysis of the prepared TLC bands after the samples were scraped and purified (fractions 1 and 2). Active fraction; 2.

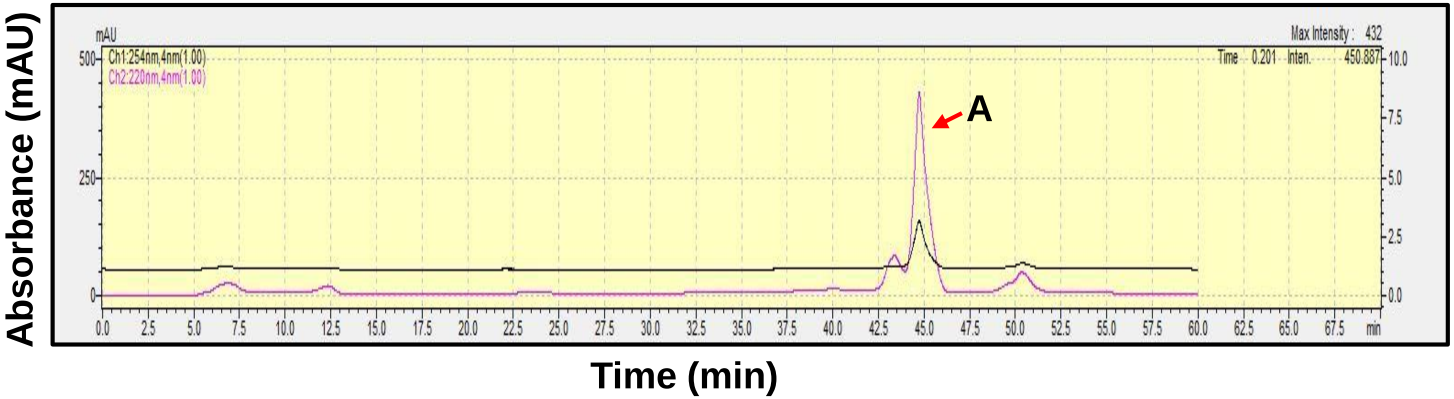

Supplementary Figure S3. Assessment of the major fractions using HPLC at two wavelengths. Samples were collected based on the 254 and 220 nm wavelengths. Active fraction; A.

A

1H NMR

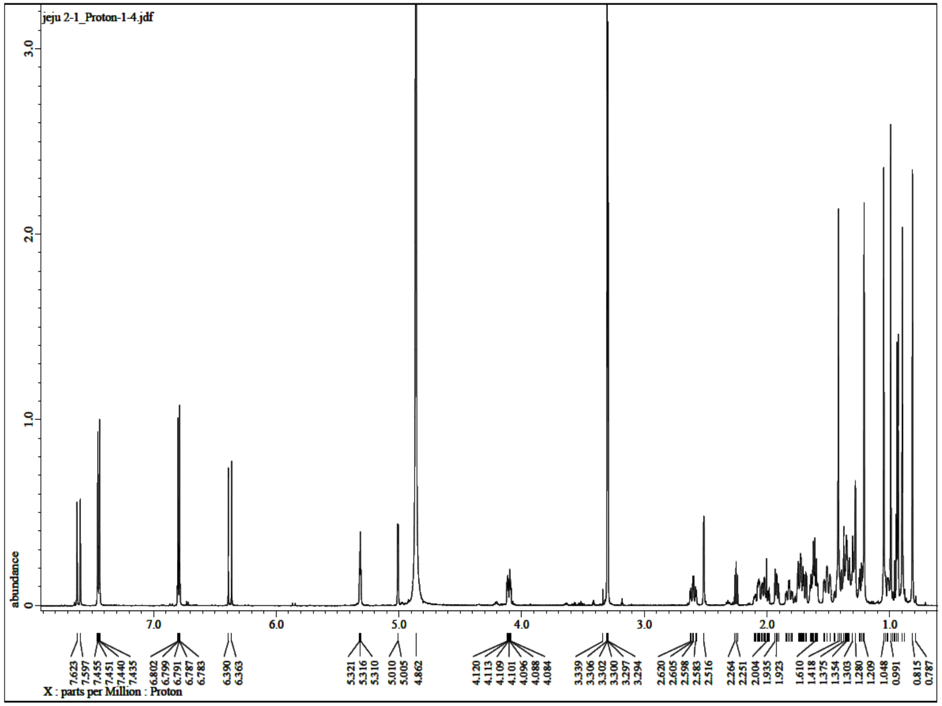

Chemical shift (ppm)

B

13C NMR

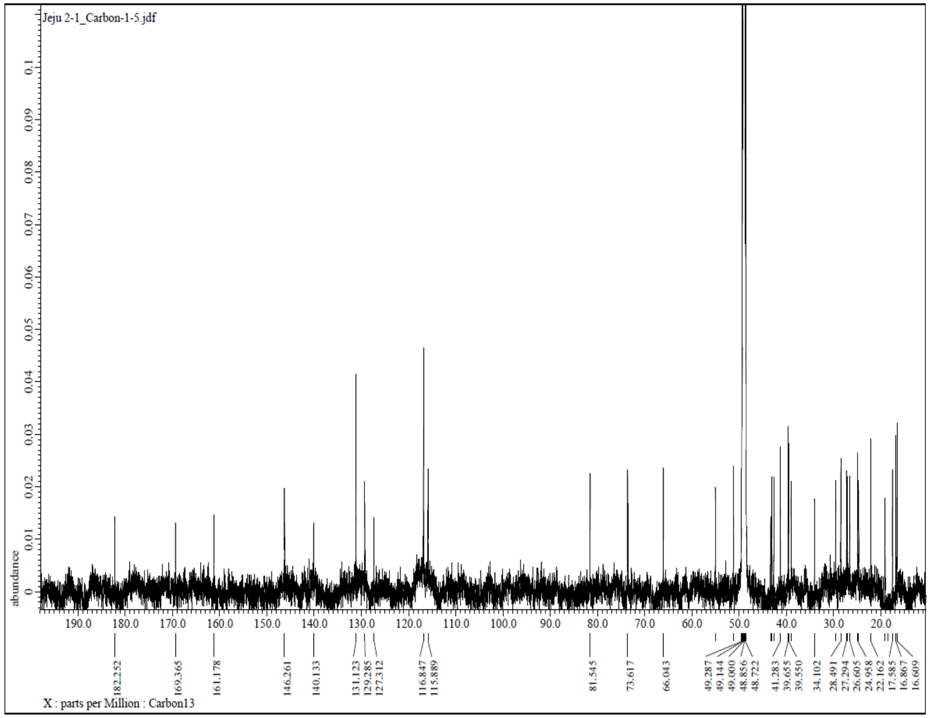

Chemical shift (ppm)

Supplementary Figure S4. 1H NMR and 13C NMR spectra of purified sample, 3-*O*-*trans*-*p*-Coumaroyltormentic acid.

A B

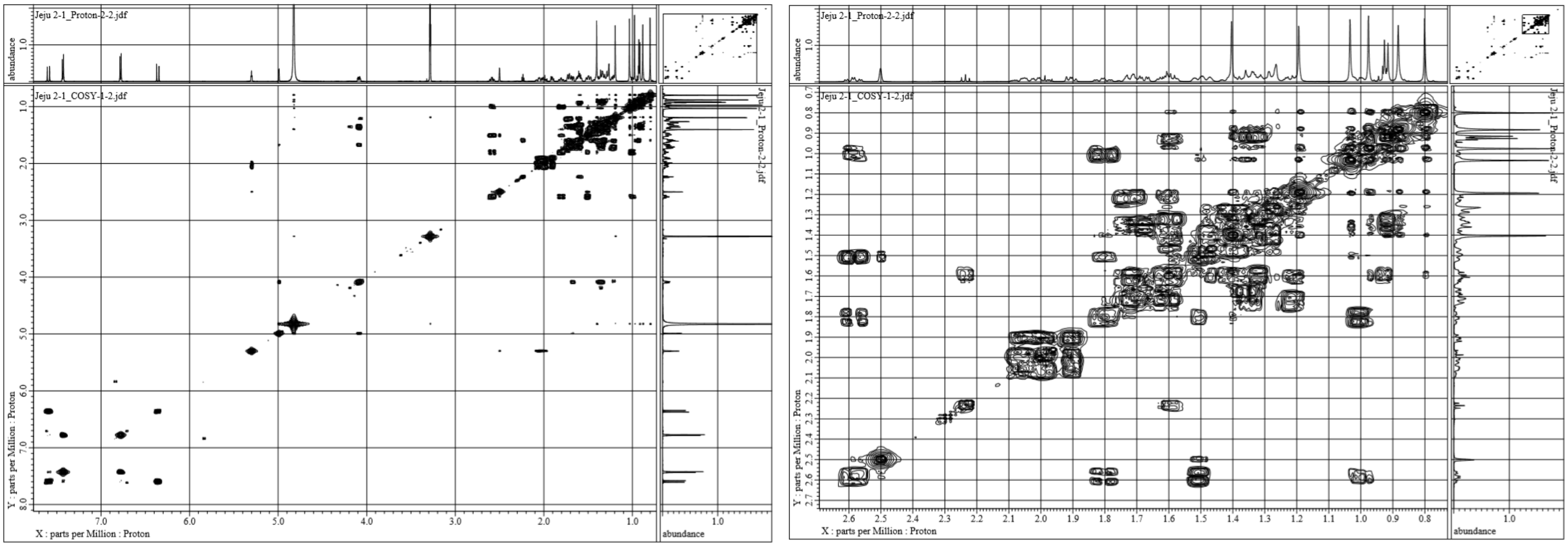

Supplementary Figure S5. COSY 2D-NMR spectra of the purified sample, 3-*O*-*trans*-*p*-coumaroyltormentic acid.

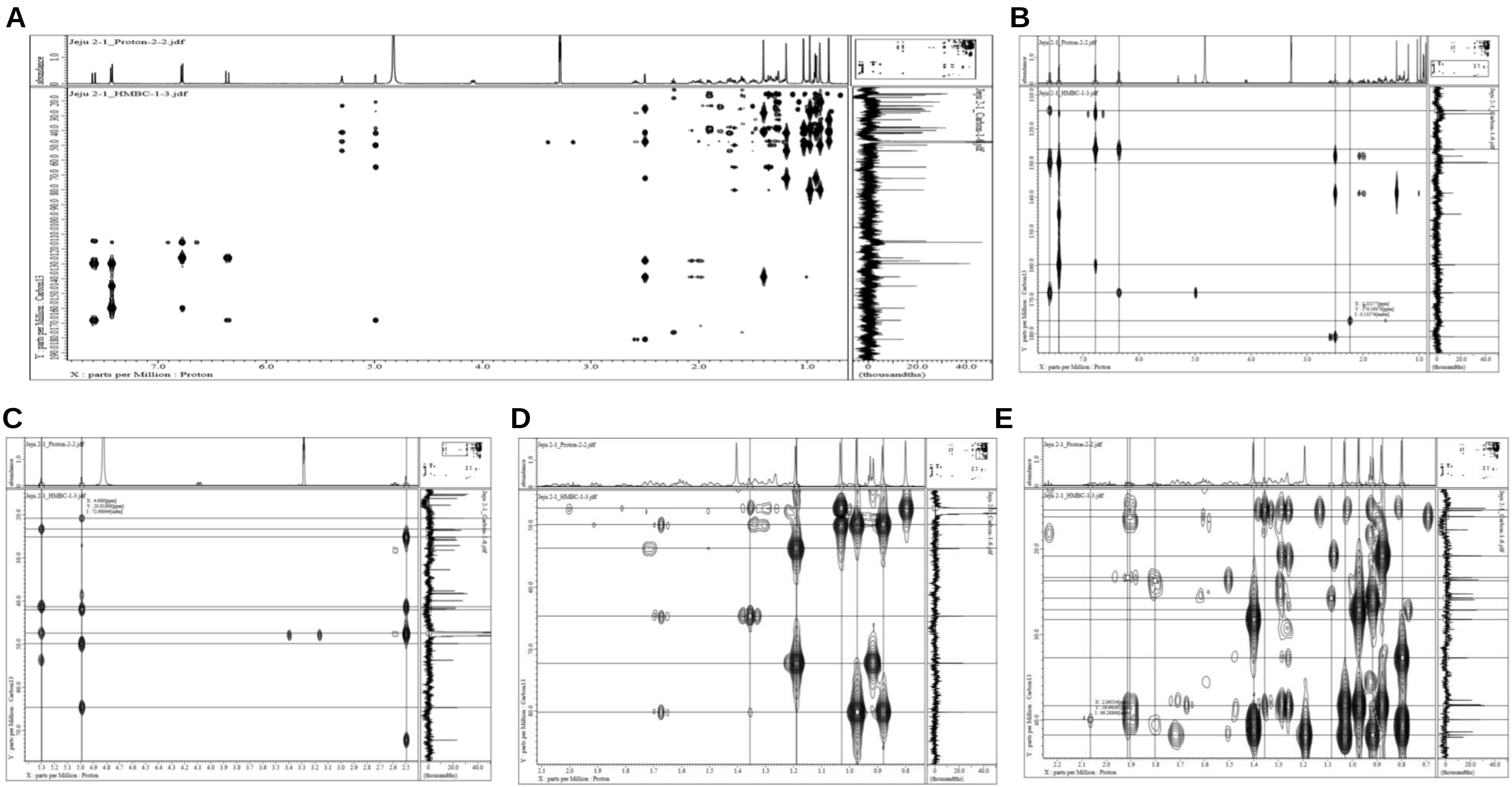

Supplementary Figure S6. HMBC 2D-NMR spectra of the purified sample, 3-*O*-*trans*-*p*-coumaroyltormentic acid.

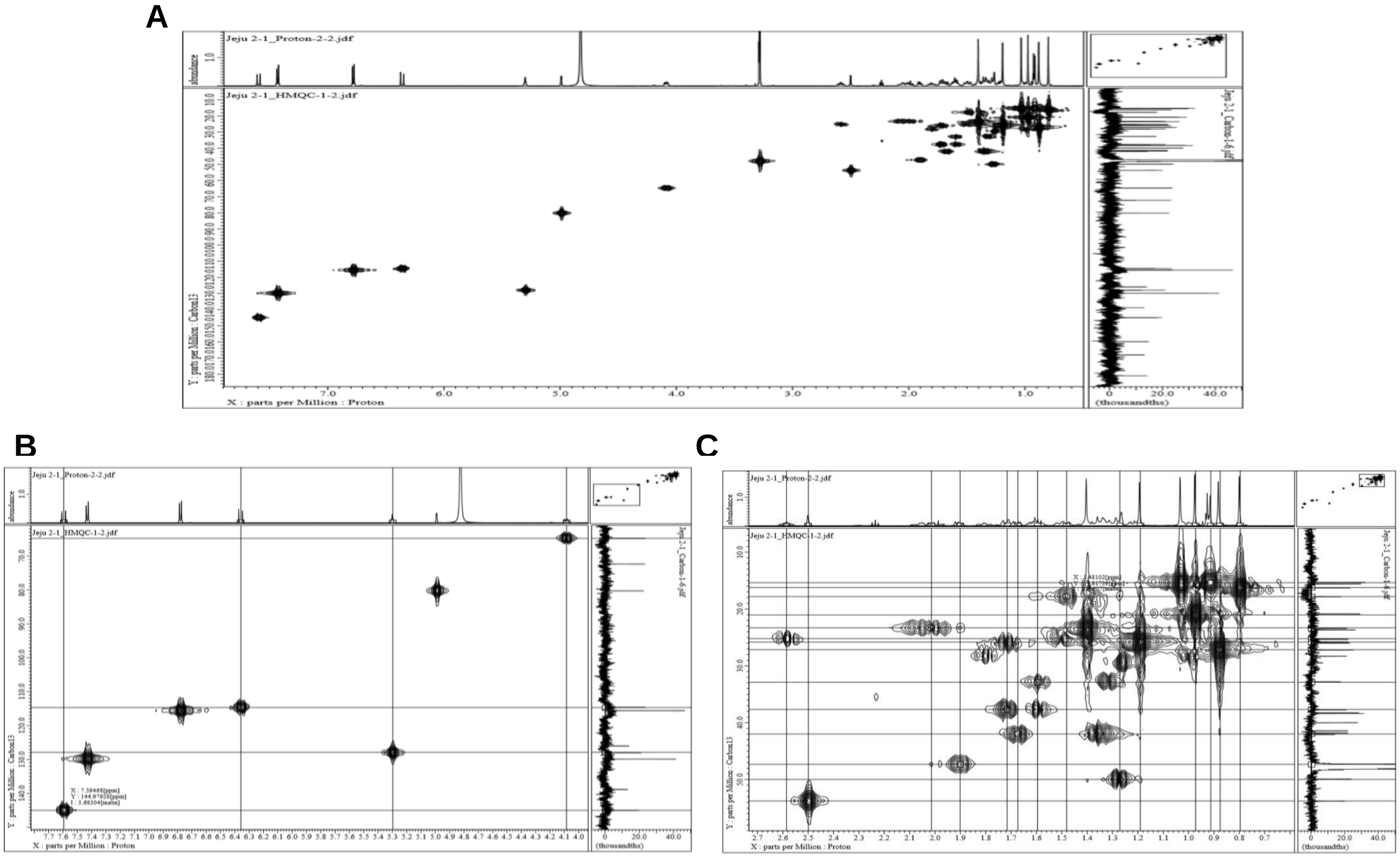

Supplementary Figure S7. HMQC 2D-NMR spectra of the purified sample, 3-*O*-*trans*-*p*-coumaroyltormentic acid.

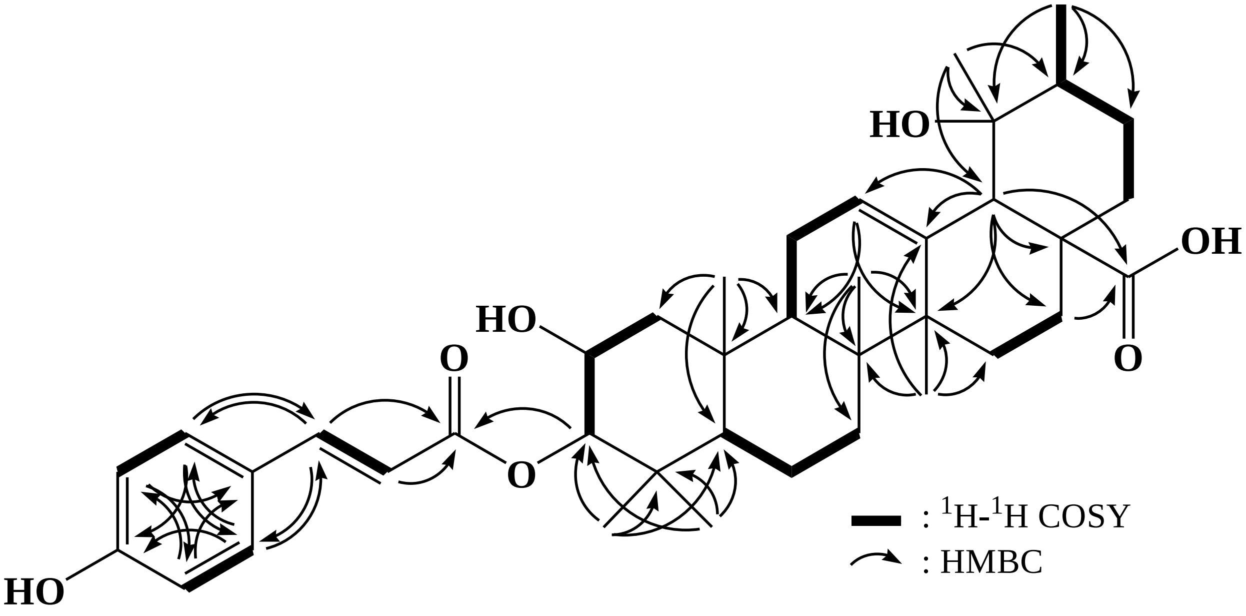

3-*O*-*trans*-*p*-Coumaroyltormentic acid: $C_{39}H_{54}O_7$, molecular weight; 634

Supplementary Figure S8. Molecular structure of the purified sample, 3-*O*-*trans*-*p*-coumaroyltormentic acid.

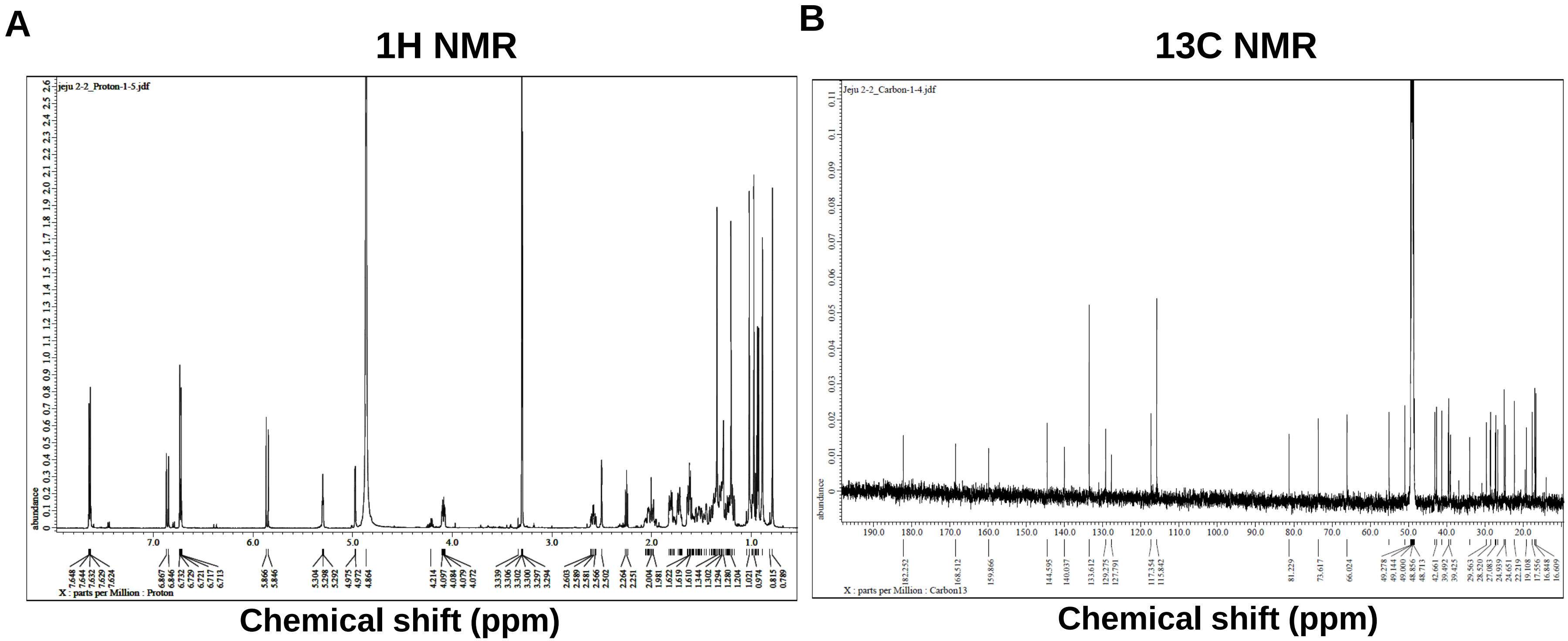

Supplementary Figure S9. ^{1}H NMR and ^{13}C NMR spectra of the purified sample, 3-*O*-*cis*-*p*-coumaroyltormentic acid.

A

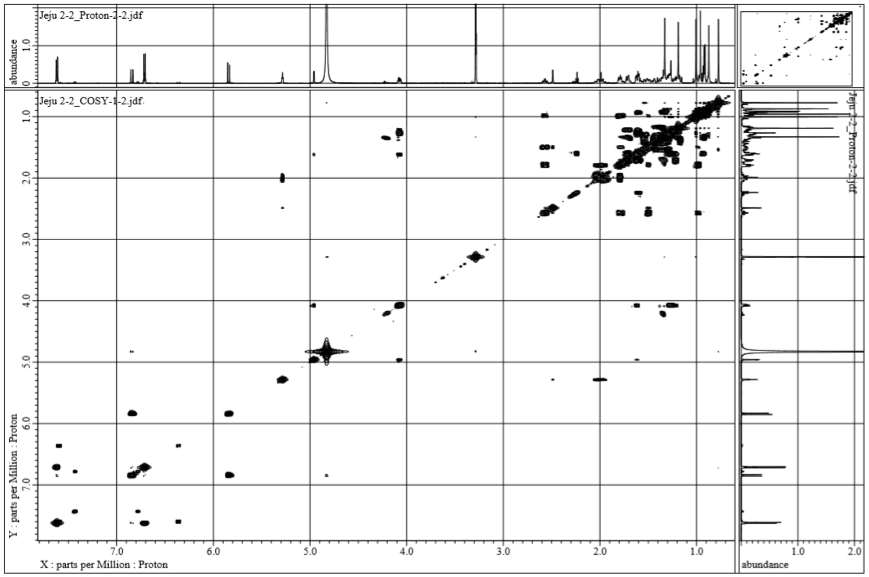

B

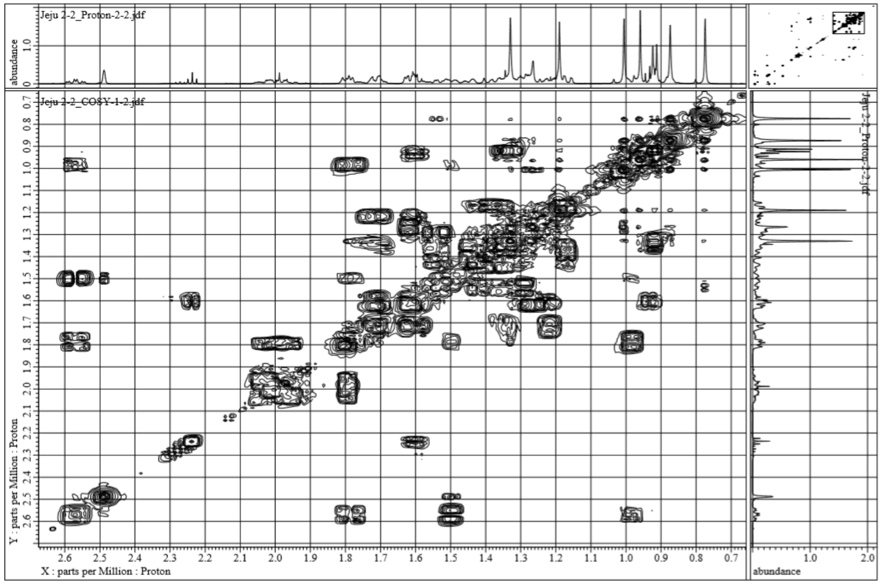

Supplementary Figure S10. COSY 2D-NMR spectra of the purified sample, 3-*O*-*cis*-*p*-coumaroyltormentic acid.

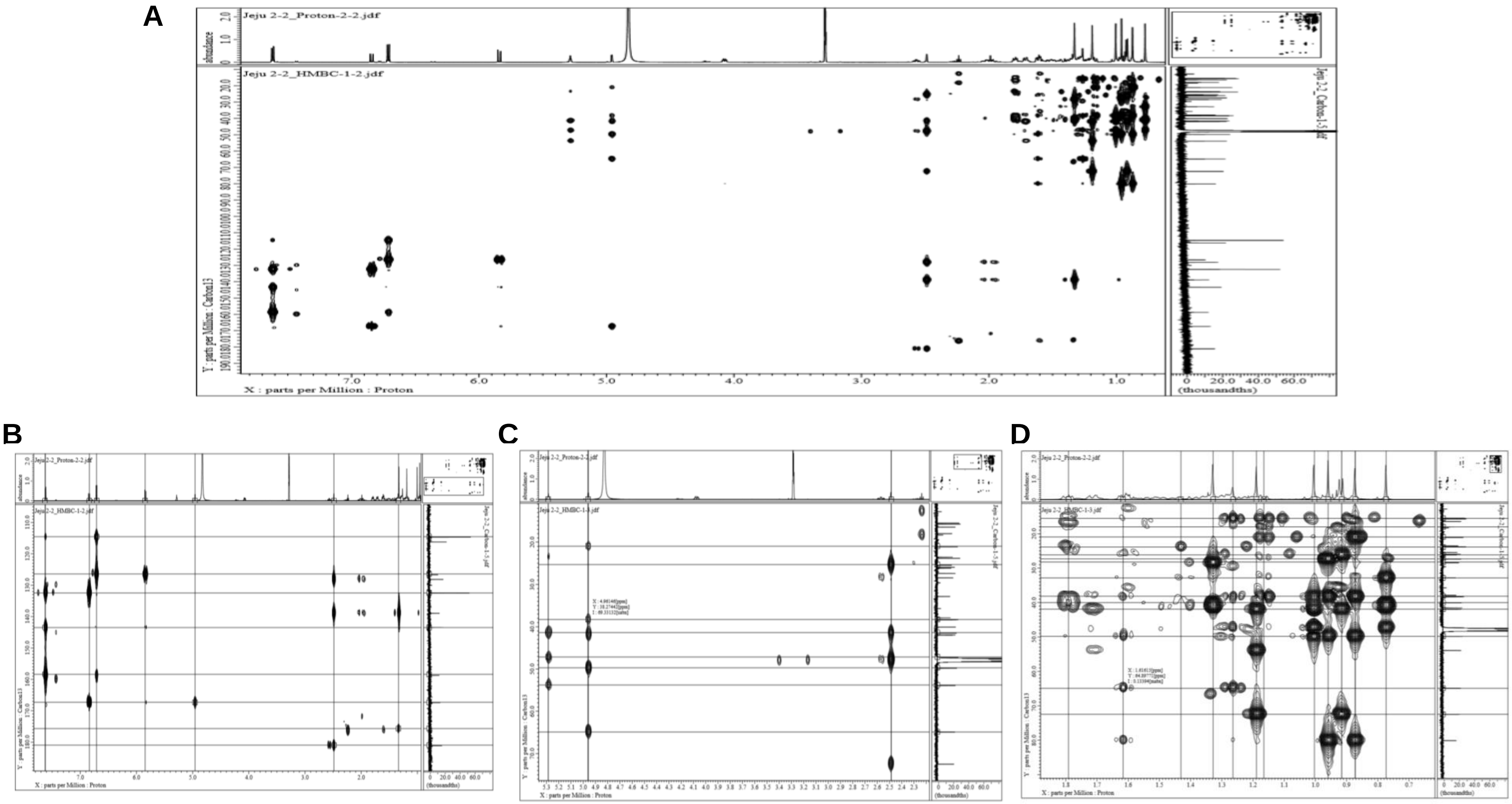

Supplementary Figure S11. HMBC 2D-NMR spectra of the purified sample, 3-*O*-*cis*-*p*-coumaroyltormentic acid.

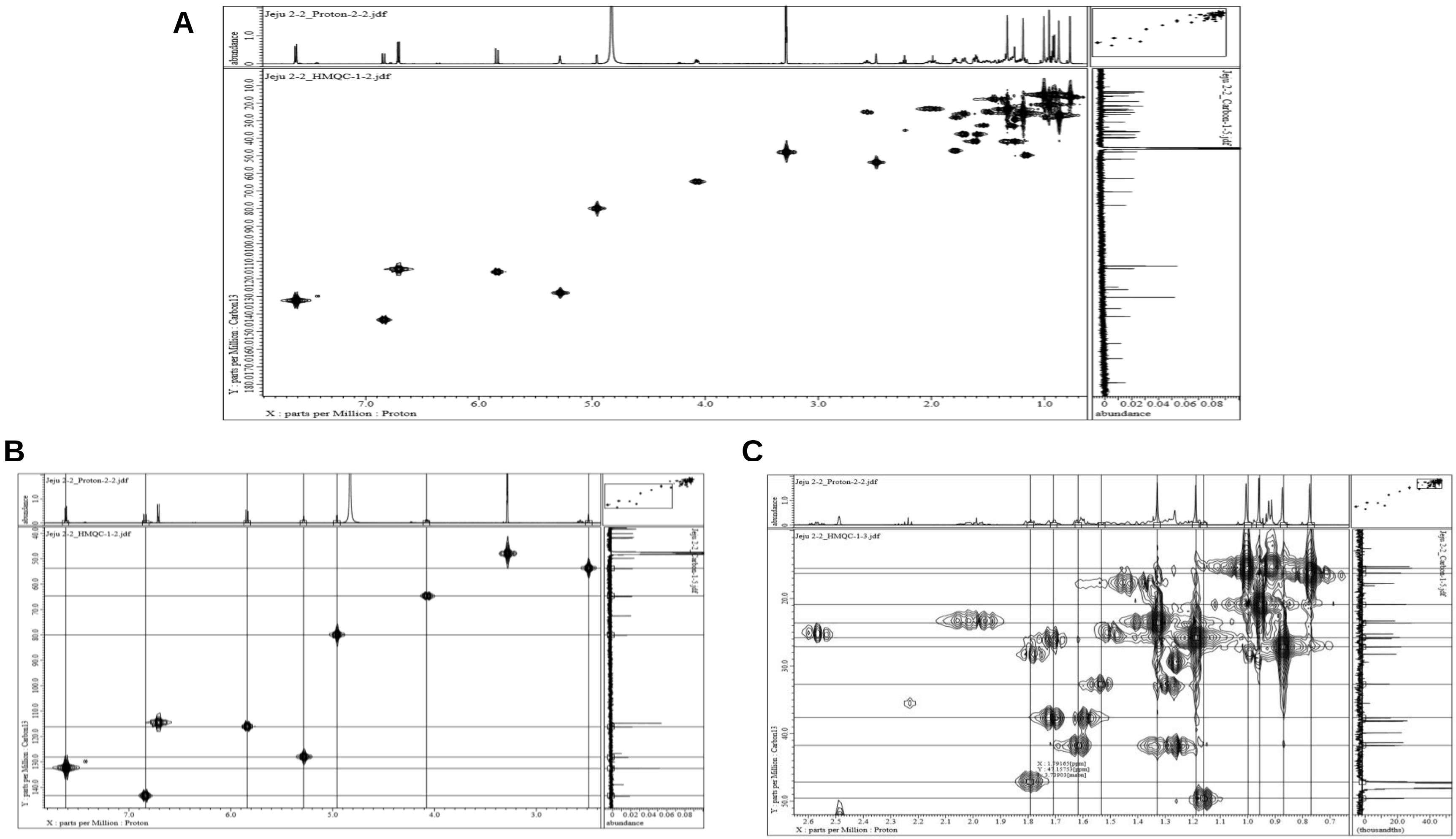

Supplementary Figure S12. HMQC 2D-NMR spectra of the purified sample, 3-*O*-*cis*-*p*-coumaroyltormentic acid.

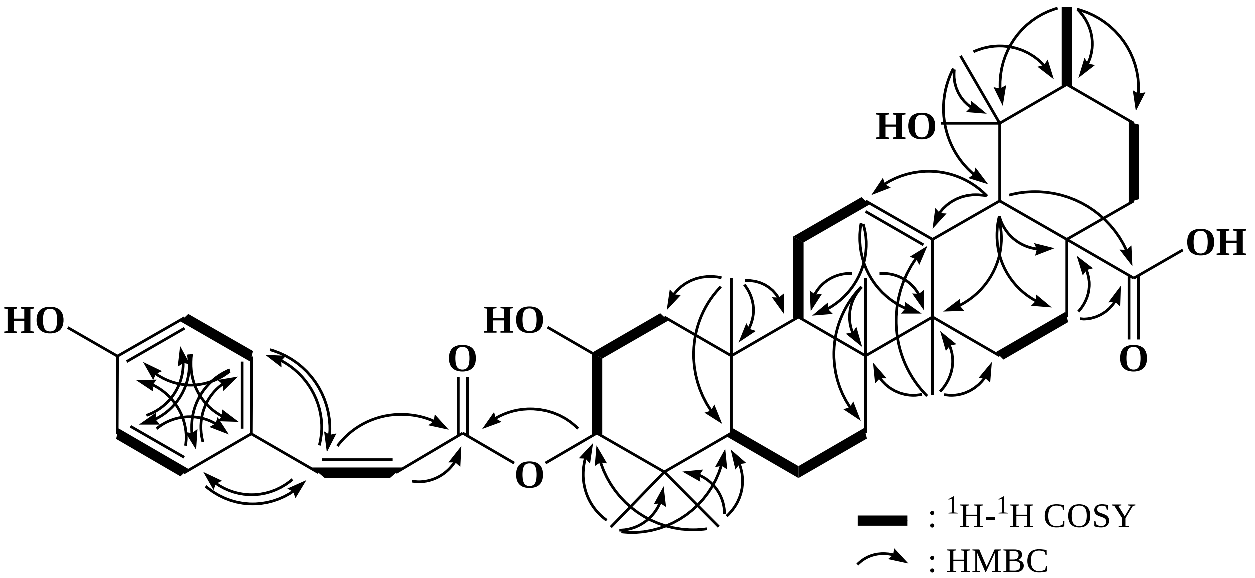

3-*O*-*cis*-*p*-Coumaroyltormentic acid: $C_{39}H_{54}O_7$, molecular weight; 634

Supplementary Figure S13. Molecular structure of the purified sample, 3-*O*-*cis*-*p*-coumaroyltormentic acid.

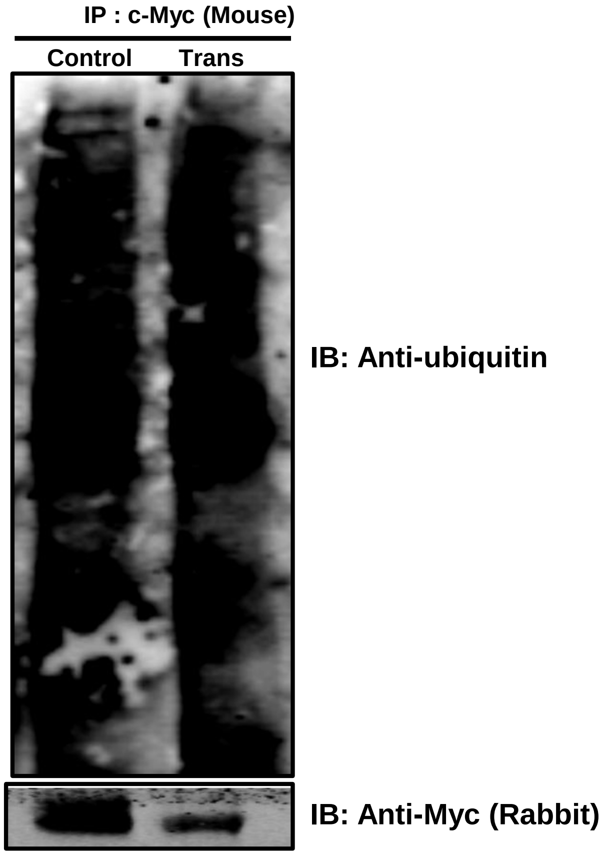

Supplementary Figure S14. 3-*O*-*trans*-*p*-Coumaroyltormentic acid (Trans) did not promote ubiquitin (Ub)-mediated proteasome degradation of c-Myc in MDA-MB-231 cells. The cells were treated with 20 μM Trans for 24 hours and then exposed to MG-132 before lysis for immunoprecipitation (IP). Cells were lysed for Western blot (WB) analysis. Control and Trans.
